# Characteristics and Outcomes of Limb Loss Support Groups

**DOI:** 10.1016/j.arrct.2025.100485

**Published:** 2025-06-20

**Authors:** Jennifer Crumling, Nicole Crumling, Prateek Grover

**Affiliations:** Penn State University College of Medicine, Hershey, PA

**Keywords:** Amputee, Limb loss, Rehabilitation, Self-help group, Support group

## Abstract

**Objective:**

To systematically understand the structure and outcomes of limb loss support groups.

**Data Sources:**

Literature review performed during June and July 2024. Search strategy included terms related to amputation and support group. Databases included PubMed, Scopus, Web of Science, CINAHL, and Cochrane Library. Publication range was 2009-2024.

**Study Selection:**

Preferred Reporting Items for Systematic Reviews and Metanalyses methodology was used to identify 7 appropriate articles of 199 initial articles. Study quality was assessed using the National Heart, Lung, and Blood Institute Scale.

**Data Extraction:**

Logic model constructs were used to extract support group inputs, outputs, and outcomes, and Expert Recommendations for Implementing Change constructs were used to extract recommendations/strategies.

**Data Synthesis:**

All 7 studies discussed inputs including participant age, sex, and race/ethnicity. Most support groups were predominantly White and younger and had more affluent attendees than the general limb loss population. Outputs were discussed in 5 studies, with location, group leadership, and other personnel being the most commonly discussed outputs. All studies discussed positive outcomes such as physical ability and mobility improvement on the Prosthetic Limb Users Survey of Mobility, emotional development with improved Posttraumatic Growth Index scores and depression symptoms, and learning and prosthesis advancement with improved self-efficacy and return to life. Six articles discussed implementation strategies including facilitation of support groups, promotion of adaptability, shadowing of other experts, and tailoring of strategies.

**Conclusions:**

These data highlight structure, development strategies, and positive effects of support groups for mobility, emotional well-being, and learning in individuals with limb loss. Furthermore, this study emphasizes the need for continued investigation into support mechanisms and formal incorporation of support groups into limb loss rehabilitation programs.

Each year, approximately 185,000 amputations are performed in the United States, with nearly 2 million people with limb loss (PwLL).^1^, ^2^, ^3^ A significant portion of these amputations result from dysvascular conditions, including vascular disease, which accounts for 82% of all major amputations,^1^ and diabetes-related complications, which contribute to 50% of lower extremity amputations.^2^ Most individuals with limb loss are over the age of 65, with an increased rate of limb loss over age 85.^4^ To support this community, there are over 400 registered support groups across the country, providing resources and assistance to those affected.^5^

Support groups hold potential benefits for PwLL by providing an environment conducive to both informational and emotional support. Within these groups, patients can address psychosocial challenges in a supportive and secure setting. Specifically for limb loss, the group setting can foster a sense of independence and optimism about the future with real-life validation of the varying phases during recovery.^6^ Support groups can be beneficial for caregivers as well, as they also experience psychological, physical, mental, and financial stresses, necessitating supplementary support mechanisms.^7^

In the absence of a well-coordinated health system to help PwLL with education, shared decision making, and system navigation, a substantial number of individuals may find their journey extremely challenging, leading to devastating effects not only on physical health but also mental health, including higher levels of anxiety, depression, and emotional stress as well as negative perceived body image.^8^, ^9^, ^10^, ^11^ Given the profound effect of amputation on PwLL, and the supportive role of support groups, there exists an opportunity to improve health outcomes for PwLL through further research and establishment of guidelines on design and implementation of sustainable support groups.

Support groups are beneficial to PwLL and their caregivers; therefore, it is important for rehabilitation programs to incorporate these programs in the care of this population. However, limb loss support group structure and outcomes are not well understood, leading to disparities in care. This scoping review aimed to systematically examine the structure and outcomes of limb loss support groups in the current literature to inform future evidence-based guidelines and support their integration into rehabilitation programs.

## Methods

A literature search for articles on limb loss support groups was conducted using scoping review methodology, and the final article set was characterized and analyzed for quality (literature selection, characterization). A data extraction framework was designed using Logic Model constructs (input, output, outcomes)^12^ to systematically abstract data from the articles on support group participant characteristics, support group characteristics, and effect of support group on participants (Logic Model-based data extraction). Finally, relevant Expert Recommendations for Implementing Change (ERIC) model strategies^13^ were explored within the selected studies to derive a literature-based collection of guidelines to create and sustain support groups (ERIC strategies exploration)

### Literature selection and characterization

The scoping review used Preferred Reporting Items for Systematic Reviews and Metanalyses model (PRISMAScr) methodology to search PubMed, Scopus, Web of Science, CINAHL, and Cochrane Library databases between June and July 2024. The search strategy was defined with a Penn State College of Medicine librarian. Search phrases varied by the database and included Medical Subject Headings or predefined thesaurus keywords, each in conjunction with self-selected keywords (support group, self-help group, encounter group, peer support, limb loss, loss of limb, amputation, amputees).

Study inclusion criteria were as follows: First, papers needed to be published in English, in the past 15 years (between 2009 and 2024). This timeframe was chosen to capture the effect of recent advancements in technology and the evolving prominence of limb loss support groups. Second, the papers had to address support groups specifically for limb loss, limb difference, amputees, or postamputation. Third, they needed to discuss aspects such as the establishment, organization, effectiveness, or outcomes of these support groups. Additionally, only high-quality and well-written papers were considered based off of the National Heart, Lung, and Blood Institute (NHLBI) scale, including original research studies, systematic reviews, and meta-analyses. Exclusion criteria included case reports, conference proceedings, and any papers with unavailable full texts.

The final article set was then analyzed in terms of article characteristics for contextual insight regarding country of origin, aims/purpose, and study design and for article quality using the NHLBI scale.

### Logic Model-based data extraction

**Fig 1:**
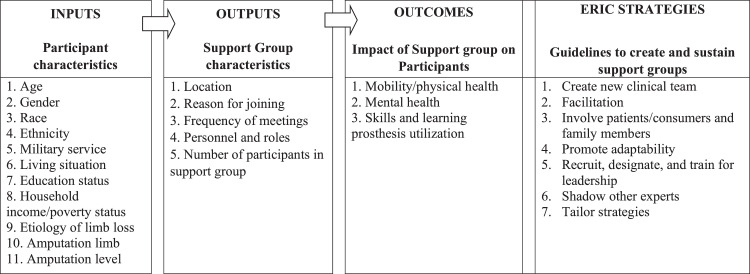
Data extraction framework based upon Logic Model and ERIC strategies: inputs, outputs, and outcomes (Logic Model) and guidelines to create and sustain support groups of support groups (ERIC strategies).

Support group participant characteristics (input) included age, sex, race, ethnicity, military service, living situation, education status, household income/poverty status, etiology of limb loss, amputation limb, amputation level (fig 1). Support group characteristics (outputs) included location, reason for joining, frequency of meetings, personnel and roles, and number of participants. The effect of support group on participants (outcomes) included mobility/physical health, mental health, skills, and learning prosthesis utilization domains.

### ERIC strategies exploration

Of 73 ERIC discrete implementation strategies, 7 were selected individually by 2 reviewers based on qualitative review of final article set. The selected strategies included (1) creating new clinical teams, (2) facilitation, (3) involving patients/consumers and family members, (4) promote adaptability, (5) recruit, (6) designate, (7) train for leadership, (8) shadow other experts, and (9) tailor strategies. The 2 reviewers then individually extracted data using this set of strategies and discussed to arrive at a consensus on article choice of strategies adopted.

## Results

### Literature selection

**Fig 2:**
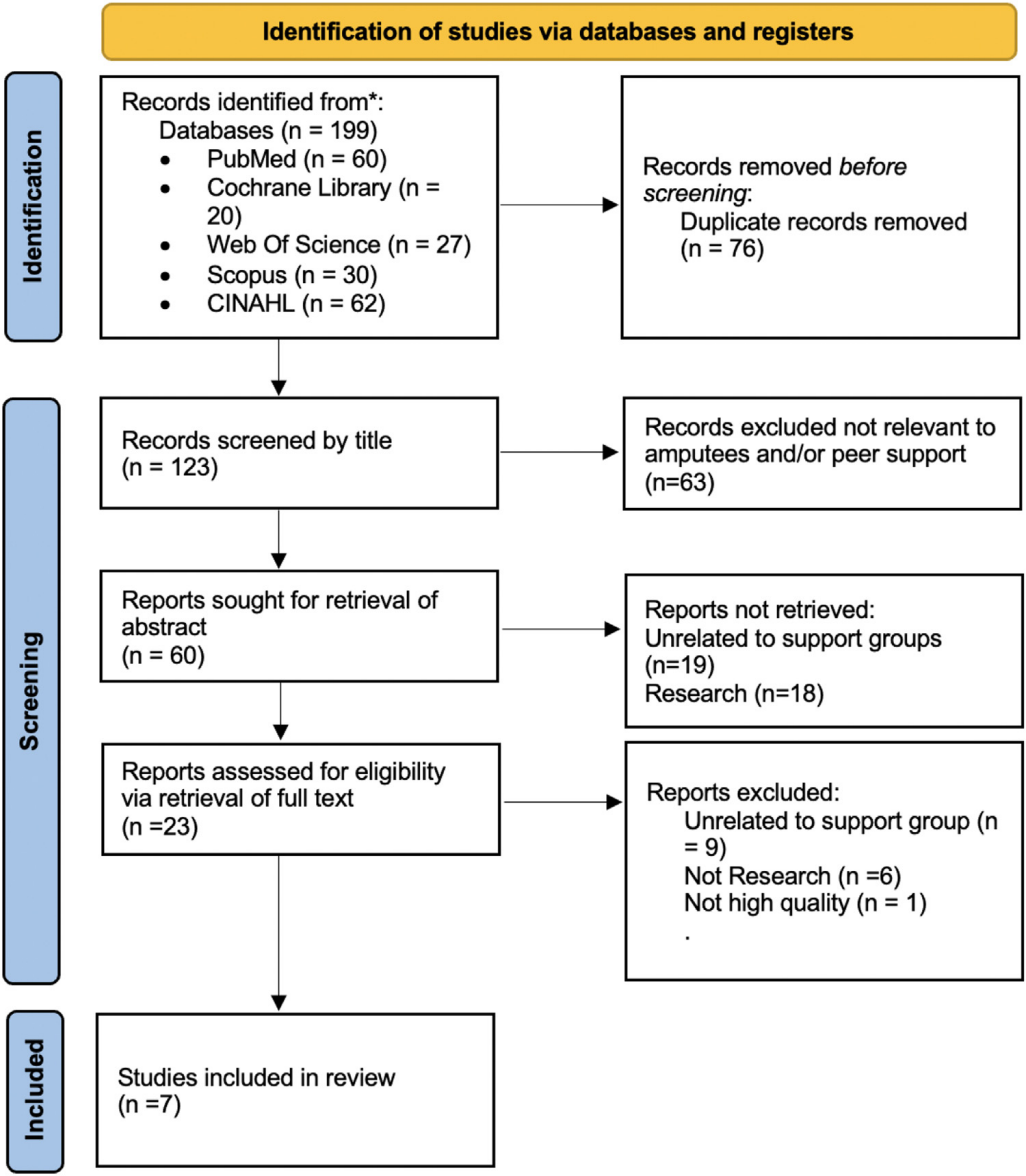
PRISMA flow diagram.

From the initial search, 199 articles were retrieved, and deduplication resulted in 123 records. Upon screening titles, 63 were excluded as they were unrelated to amputees and/or peer support. Of the remaining 60, the abstract search excluded 37 as they were unrelated to support groups (n=19) or research (n=18). The 23 remaining article full texts were screened for eligibility by 2 reviewers independently, and 16 were excluded as not being relevant research (n=6) and for quality (n=1), leaving 7 studies for data (fig 2).

### Brief description of included studies (n=7)

Nathan and Winkler^14^ conducted an online survey study that found that the needs of participants in amputee support groups change over time, meeting content must be relevant to participants, participation in a support group is driven by desire to improve functionality, and technology use should support patient needs within a support group. Specifically, regarding the use of technology, Nathan and Winkler^14^ found that most amputees that completed the surveys believed audio and video teleconferencing or avatar-based technology would improve participation in support groups for limb loss.^14^

Keeves et al^15^ performed an exploratory qualitative survey study that found people after traumatic lower limb amputation may benefit from peer support groups in adjusting to disability and community participation through retraining skills and psychosocial support. For example, people with traumatic lower limb amputation identified inaccessibility of the built environment and prosthetic mobility physical challenges to be hardships to participation in daily life.

Stutts and Stanaland^16^ led an online survey study on posttraumatic growth in traumatic amputations in veterans and civilians, which found that consistent engagement in support groups are associated with higher levels of posttraumatic growth via posttraumatic growth index (PTGI) scores.

Lee et al^17^ performed a quantitative and qualitative descriptive survey study that found that persons with lower limb loss had positive experiences with peer support activities in terms of informational and emotional support. Peer support also led to improved mobility, with participant scores increasing to the 55th from the 36th percentile on the Prosthetic Limb Users Survey of Mobility (PLUS-M) ^17^.

Wegener et al^18^ conducted a randomized control trial compared the acceptance and effectiveness of a community-based self-management intervention with that of community-based support groups. Furthermore, this study found that the self-management intervention improved emotional and physical components after treatment better than the community-based programs, although both interventions were advantageous.^18^

Brusco et al^19^ performed a cost-effectiveness analysis on the amputee peer support program, which found that peer support for PwLL had a positive effect on both those receiving and those providing support and is an inexpensive addition to care. Peer support groups for PwLL were found to provide information, social support, and a sense of understanding to participants.^19^

Costa-Parke et al^20^ completed a scoping review to identify evidence, definitions, and key factors related to peer support for PwLL and found that research on this subject is limited, with only 2 randomized controlled trials identifying the effects on physical functioning and 4 studies identifying benefits of psychological well-being. More research is necessary to evaluate the effectiveness and tailor interventions for support groups for PwLL.^20^

### Study quality and characteristics

**Fig 3:**
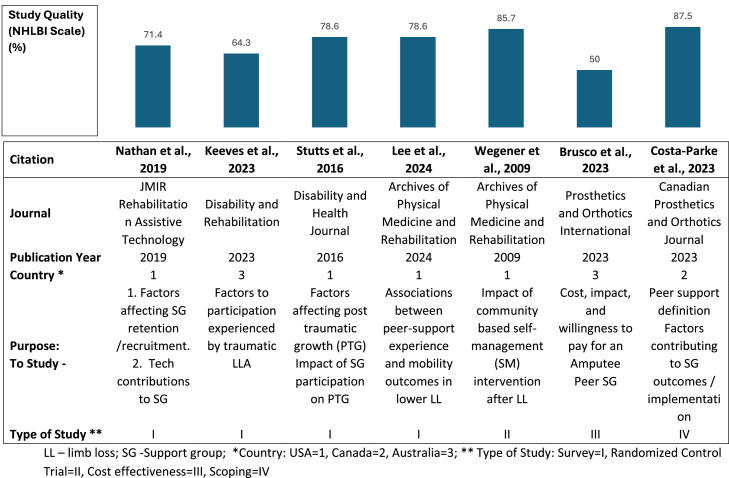
Study characteristics and quality.

Of the final set, 4 studies were from the United States,^14^^,^^16^, ^17^, ^18^ 1 from Canada,^20^ and 2 from Australia.^15^^,^^19^ The study type included 4 surveys,^14^, ^15^, ^16^, ^17^ 1 randomized control trial,^18^ 1 cost analysis,^19^ and 1 scoping review.^20^ Quality assessments scores (0-14 scale, higher is better) for the survey studies ranged from 9^15^ to 11^16^^,^^17^ of 14, highest for the randomized control trial (12/14)^18^ and lowest for the cost analysis (7/14).^19^ The scoping review was graded on an 8-point scale based on the NHLBI quality assessment of systematic reviews and meta-analyses as a 7 of 8 ^20^ (fig 3).

### Inputs: participant characteristics

All 7 articles examined the inputs of the support group (table 1). Participant age was reported in 6 studies,^14^, ^15^, ^16^, ^17^, ^18^, ^19^ with most participants aged 50-60 years,^15^, ^16^, ^17^, ^18^, ^19^ while 1 study^14^ included participants aged 20-39 years. Sex was discussed in 6 articles^14^, ^15^, ^16^, ^17^, ^18^, ^19^ with 5 reporting a predominance of male participants^14^, ^15^, ^16^^,^^18^^,^^19^ and 1 including an LGBT demographic.^14^ Four studies addressed race and ethnicity, noting a majority of White participants.^14^^,^^16^^,^^17^^,^^18^ Additional social determinants of health discussed include employment status,^16^ health insurance,^18^ and gentrification.^19^ Two studies assessed military service history, finding that most participants had no military background.^14^^,^^16^ Two studies investigated living situation, with 18.2% and 22.2% participants living alone.^15^^,^^16^ Education level was reported for participants in 3 of the studies^15^^,^^16^^,^^18^ and household income status/poverty status in 2 studies.^16^^,^^18^

**Table 1:** Support group participant characteristics.

Support Group Participant Characteristics		Nathan and Winkler^14^ (2019)	Keeves et al^15^ (2023)	Stutts and Stanaland^16^ (2016)	Lee et al^17^ (2024)	Wegener et al^18^ (2009)*	Brusco et al^19^ (2023)	Costa-Parke et al^20^ (2023)
**Age (y)**	Mean			53.6		SG 56.9; SM 55.5	57.9	
	Range	20-39	50-64	23-82	35-66+			
**Sex (%)**	Male	65	77.8	54.5	47	SG 59; SM 55	68	
	Female	33	22.2	45.5	53	SG 41; SM 45	32	
**Race (%)**	Caucasian	93		86.4	80.7	SG 78.9; SM 78.2		
	Black	6		6.1	7.2	SG 11.9; SM 32		
	Native American	2						
**Ethnicity (%)**	Hispanic	4		4.5	3.6	SG 4.4; SM 21		
	Non-Hispanic	96			91.6			
**Military service (%)**	Military	15		22.7				
	Non-Military	85		77.3				
**Living situation (%)**	Independent		22.2	18.2				
	With others		77.8	78.8				
**Education status (%)**	High school		55.6	12.1		SG32.5; SM 29.1		
	Further education		44.4	88		SG 67.4; SM 70.9		
**Household income status/ poverty status (%)**				Income level under $15000 -10.6, $15000 to <$25000 - 9.1, $25000 to <35000 - 3.0, $35000 to <50000 - 12.1, Over $50000 - 65.2		SG: Not poor 61.8, Near poor 23.5, Poor 14.8; SM: Not poor 65.2, Near poor 22.5, Poor 12.3		
**Other social determinants (%)**				Employment status: Full time 27.3, Part time 13.6, Seeking work 7.6, Retired 33.3, Unable to work 12.1		Health Insurance- SG: Medicare 45.4, Medicaid 5.7, Private 39.2, Uninsured 4.9; SM: Medicare 45.5, Medicaid 5.8, Private 37.5, Uninsured 6.2	Metropolitan 84.6, Rural and Regional 15.4	

⁎ SG, Support Group Control; SM, Self-Management Treatment Group.

Etiology of amputation was identified in 5 articles^14^, ^15^, ^16^, ^17^, ^18^ including dysvascular (43.3±6.8, n=4),^14^^,^^16^^,^^17^^,^^18^ traumatic (45.3±30.7, n=5),^14^, ^15^, ^16^, ^17^, ^18^ and oncologic (7.3±3.3, n=3) causes.^14^^,^^17^^,^^18^ One study^15^ focused specifically on traumatic etiologies such as motor vehicle collisions and workplace accidents. Dysvascular sources were predominant in most studies^14^^,^^16^^,^^17^ with traumatic causes being more common in 2 studies.^15^^,^^18^ The amputated limb was reported in 6 articles^14^, ^15^, ^16^, ^17^, ^18^^,^^20^ with the most^14^^,^^16^^,^^18^^,^^20^ or all^15^^,^^17^ participants having lower limb amputations (90.5±11.2, n=4). Of these, 3 studies^14^^,^^15^^,^^17^ further identified participant amputation level as transtibial (64.4±7.6, n=2), transfemoral and knee disarticulation (24.0±7.0, n=2), transradial (mean=14, n=1), and transhumeral and elbow (mean=6, n=1) (table 1).

### Outputs: support group characteristics

Outputs of the support group were discussed in 5 articles (table 2).^14^^,^^17^, ^18^, ^19^^,^^20^ The location of the support group meetings was identified in 4 articles,^14^^,^^17^^,^^19^^,^^20^, with 2 identifying in-person locations,^14^^,^^20^ 1 discussing virtual meetings,^20^ and 3 discussing support groups conducted within rehabilitation programs.^17^^,^^19^^,^^20^

**Table 2:** Inputs: participant limb loss characteristics.

Participant Limb Loss Characteristics		Nathan and Winkler^14^ (2019)	Keeves et al^15^ (2023)	Stutts and Stanaland^16^ (2016)	Lee et al^17^ (2024)	Wegener et al^18^ (2009)	Brusco et al^19^ (2023)	Costa-Parke et al^20^ (2023)	Mean	SD	n
Etiology (%)	Dysvascular	44		51.5	42.7	34.8			43.3	6.8	4
						*37.1					
	Trauma	28	100	31.8	30.5	36.1			45.3	30.7	5
						*39.3					
	Oncology	6		12.1	6.1	4.9			7.3	3.3	4
						*7.3					
Amputation limb (%)	Upper	20	100	87.9		95.1		15.9	63.8	42.1	5
						*92.7					
	Lower	78	100		100			84.1	90.5	11.2	4
Amputation level (%)	Transtibial	59			69.8				64.4	7.6	2
	Transfemoral + knee disarticulation	19			28.9				24.0	7.0	2
	Transradial	14							14	N/A	1
	Transhumeral + elbow disarticulation	6							6	N/A	1

Abbreviation: N/A, not applicable.

* Indicates self management group.

The reason for joining a support group was discussed in 2 articles^14^^,^^17^ with 1 article^14^ indicating that most participants joined for learning, while another^17^ found that most participants joined for companionship and helping others (mean=52.5) and many join to enhance their learning (mean=45.1).

The frequency of support group meetings was discussed in 3 articles,^14^^,^^17^^,^^18^ all indicating that most participants attend at least monthly sessions (mean=75.4) versus less than once a month (mean=17.6).

Five articles discussed the support group leaders and other involved personnel,^14^^,^^17^, ^18^, ^19^, ^20^ identifying an experienced amputee as a key figure in all,^14^^,^^17^, ^18^, ^19^, ^20^ with 2 articles noting the involvement of health care professionals^14^^,^^18^ and 3 articles highlighting the role of volunteers^18^, ^19^, ^20^ (table 2).

### Outcomes: effect of support group on participants

All 7 articles evaluated outcomes of support groups for amputees. Three studies^17^, ^18^, ^19^, ^20^ reported positive effects of support groups on physical ability and mobility. Lee et al^17^ revealed that participants in peer support group exhibited a higher mobility score (PLUS-M T score of 51.4) compared to those without a support group (PLUS-M T score of 46.8). Furthermore, Wegener et al^18^ and Costa-Parke et al^20^ noted that participants in support groups experienced fewer functional limitations than those without support group involvement, even 6 months posttreatment (table 3).

**Table 3:** Outputs and outcomes of the support groups.

NOTE. STATISTICS (n)#: frequency, except where stated.

^⁎^ Location: In person= 1, virtual=2, rehabilitation program=3.

^†^ Support Group Personnel: health care personnel=1, experienced amputees=2, other volunteers=3.

The emotional effect of support groups on amputees was discussed in 5 studies.^16^, ^17^, ^18^, ^19^, ^20^ Stutts and Stanaland^16^ reported improvements in PTGI scores among support group participants, with a PTGI score of 66.2 compared to 45.4 for nonparticipants. Costa-Parke et al and Wegener et al highlighted reduction in symptoms^20^ and likelihood of depression.^18^ Companionship and understanding were discussed in 3 articles,^17^^,^^19^^,^^20^ with Costa-Parke et al^20^ noting that of the reviewed 7 articles indicated the development of friendships and community. Lee et al^17^ found that 44% of participants joined support groups for companionship. Specifically, Brusco et al^19^ found that 42% of patients that participated in the amputee peer support program felt that they had better access to an organization that understood their experience. Positive cognition was discussed in 2 articles,^18^^,^^20^ with 1 study indicating increased optimism and hope for the future^20^ and another discussing Positive States of Mind measurements improving at 6 months^18^ (table 3).

Learning about prosthetics and new amputee skills was discussed in 6 studies.^14^^,^^15^^,^^17^, ^18^, ^19^, ^20^ Specifically, Keeves et al^15^ reported that peer support networks helped participants return to life roles and provided advice to assist with daily tasks and community participation. Furthermore, Costa-Parke et al^20^ indicated that 2 studies explored acceptance and adaptation to major limb loss, and 1 study reported observing other amputees’ success put their experiences into perspective. Wegener et al^18^ emphasized that guided self-management in support groups resulted in higher general self-efficacy immediately posttreatment and after 6 months. Three studies^14^^,^^17^^,^^19^ identified information acquisition as a key reason for joining, with Lee et al^17^ quantifying that 27% of participants sought information on coping with amputation and Nathan and Winkler^14^ noting that 13.5% joined for relevant topics (table 3).

### Implementation: fit with ERIC model strategies

Six studies^14^^,^^15^^,^^17^, ^18^, ^19^, ^20^ discussed at least one of the ERIC model strategies was used. Creating new clinical teams was discussed in 3 articles,^14^^,^^18^^,^^20^ with Wegener et al^18^ comparing typical support groups to self-management-based support groups. Facilitation of support groups was covered in 6 articles.^14^^,^^15^^,^^17^, ^18^, ^19^, ^20^ Involving patients/consumers and family members was discussed in 3 articles,^17^^,^^18^^,^^20^ highlighting the importance of patient roles^18^^,^^20^ and peer involvement.^17^ The strategy to promote adaptability was identified in 4 studies,^14^^,^^15^^,^^18^^,^^19^ with Nathan and Winkler^14^ identifying technology as a means to simplify support group attendance through teleconferencing and virtual reality. Brusco et al^19^ discussed the cost of participation and patient willingness to pay. Only 1 article discussed the ERIC model strategy to recruit, designate, and train for leadership. Wegener et al^18^ described that the 2 groups in the trial had trained leadership. Four articles^14^^,^^15^^,^^17^^,^^20^ discussed the strategy to shadow other experts, with 3 of the articles^15^^,^^17^^,^^20^ highlighting the role of amputees in supporting other amputees. Tailoring strategies was discussed in 4 articles,^14^^,^^15^^,^^17^^,^^20^ with all 4 identifying significant barriers to amputee support groups, particularly related to mobility and accessibility^14^^,^^15^ (table 4).

**Table 4:** ERIC model.

NOTE. Strategy described: yes (black cells), no (gray cells).

^⁎^ See Powell et al.^13^

## Discussion

This scoping review on support groups for limb loss utilizes implementation science methodology to systematically analyze the rather limited literature for inputs, outputs, outcomes, and implementation strategies. Together, this framework and data provide metrics and benchmark data for health care organizations seeking to amplify their community service mission through development and/or refinement of limb loss support groups.

### Study selection and characteristics

Only 7 studies met the criteria for inclusion in this review, a significantly lower number compared to studies focused on support groups for other medical conditions, such as HIV and AIDS (n=22),^21^ online support groups for family caregivers caring for a variety of patient conditions (n=19),^22^ and acquired brain injury (n= 17).^23^ This paucity of research on limb loss support groups is particularly concerning as it limits the ability to identify key facilitators, barriers, and the overall effectiveness of these groups, thereby hindering efforts to establish consistently effective support groups.

### Inputs: limb loss characteristics

The study sample was representative of the current population, with dysvascular etiologic predominance (43.3±6.8%, n=4) consistent with literature that describes 50% for lower extremity amputations as complications of diabetes,^2^ and 82% of all major amputations as a result of vascular disease.^1^ Similarly, this study’s lower limb predominance (90.5±11.2%, n=4) amputation is consistent with literature describing upper limb amputation noted in 8%,^3^ which highlights the need for support for upper limb loss as there is decreased prevalence with likely less support and education.

### Inputs: participant characteristics

Age was discussed in 6 studies, with most participants in the 50s^15^^,^^16^^,^^18^^,^^19^; however, 1 article^14^ had younger participants aged 20-39 (table 1). This population differs from reports showing that there are higher rates of limb loss in the population over age 85.^4^ This difference in participation may be because of older populations having less willingness and accessibility to participate in research studies and attend meetings. Men and women were included in all the studies, which is indicative of the general population of amputees.^14^, ^15^, ^16^, ^17^, ^18^ Race and ethnicity were reported in 4 of the included studies with a predominantly non-Hispanic White population.^14^^,^^16^^,^^17^^,^^18^ This study population is more reflective of the local racial and ethnic makeup and does not reflect that non-White race/ethnicities have amputations at higher rates according to reports in 2014.^24^ A recent US Government Accountability Office report highlights that approximately 66% of Medicare beneficiaries with amputations are White; however, these same reports acknowledge that African Americans were disproportionally affected by limb loss in 2024 than other ethnicities.^25^ Of the Medicare beneficiaries who lost a limb in 2016, 21% were Black, while only 8% of all Medicare beneficiaries overall were Black.^25^ Additionally, 9.3% of Medicare beneficiaries with limb loss in 2016 were Hispanic.^25^ This discrepancy may highlight the lack of support extended to minority populations as well as potential biases, which may lead them to not trust the health system and hence not participate in support groups, even if they have access.^26^ For example, Angelo et al^26^ found that 43% of African Americans had medical distrust, which was significantly greater than that of the White population of 35%. This higher level of medical distrust also emphasizes the inequity leading to amputations because of dysvascular etiologies and complications.^24^ Household income or poverty level was reported in 2 studies, revealing that the most participants have a household income of over $50,000^16^ and an income level of “not poor”^18^. This may imply that people with a lower median household income, which is also associated with a higher odds ratio for amputation over all insurance types (Medicare, Medicaid, private, and uninsured), may not attend support groups. The difference in income and income level between the study population and general PwLL population can be attributed to time, support, travel, and education constraints leading to difficulties becoming involved in support groups.

Understanding these patient demographics and amputation characteristics is essential for organizations, as these elements affect participants’ comfort levels and the sense of camaraderie within the group. The reasons that patients may or may not join support groups can reveal gaps in patient knowledge acquired through conventional clinical practices, highlight their specific learning needs and values, and guide support group design to better meet participants’ needs and enhance their experience.

### Outputs: support group characteristics

Rehabilitation programs were the commonest setting for support groups, followed by in-person, and virtual was least favored. This diverse support group meeting format is similar to a national amputee peer support program by Hanger Clinic called AMPOWER that facilitates its peer visits through multiple platforms as preferred by the amputee.^1^ The choice of meeting format is particularly significant for PwLL, who may encounter unique mobility challenges that make in-person meetings difficult to attend. Conversely, some individuals may lack the technological proficiency required for virtual formats, which can affect their comfort and participation. Unique to support groups for limb loss, there are discussions of incorporating virtual reality^14^ as well as the role for support groups in the rehabilitation programs,^17^^,^^19^^,^^20^ whereas research on support groups for other populations such as stroke and cancer support groups discuss the role of one-on-one sessions.^27^^,^^28^. Incorporating support groups into rehabilitation programs can mitigate accessibility issues and reduce technological barriers because patients are already present at the location. However, this format is not a long-term solution, as patients eventually transition out of rehabilitation and must adapt to regular life circumstances.

Regarding leadership, this review found consensus on including experienced PwLL in support group leadership roles. While other research recognizes the value of lived experiences, it is unclear whether an experienced person should serve as a leader or as supplemental personnel.^28^ PwLL as leaders can share unique experiential insights that those without limb loss may not be able to offer, facilitating more effective support group interactions. This is seen in a conceptual framework involving peer mentorship, providing guidance and companionship to a cohort of upper limb amputees.^6^ Furthermore, research on other support groups debates the role of health professionals leading or supplementing the group, which contrasts with this review’s stand for the substantial role of health professionals, with no negative effect found.^14^^,^^20^ This view is further supported by a study of stroke support groups in which health professionals provided “a professional viewpoint” while simultaneously providing a learning experience for PwLL.^27^

### Outcomes: effect of support group on participants

This review found that all eligible studies reported benefits associated with support groups for PwLL. This observation aligns with a broader body of research indicating that support groups tailored to specific medical conditions consistently enhance social integration, alleviate symptoms, support decision making, and promote emotional well-being, thereby playing a crucial role in improving overall quality of life across various patient populations.^27^, ^28^, ^29^, ^30^ Despite the limited research and lack of standardized guidelines for limb loss support groups, the evidence suggests that these groups are effective in addressing the needs of individuals with limb loss. With appropriate guidance and implementation strategies, limb loss support groups have the potential to become even more effective and beneficial than what is currently documented.

Notably, this review highlighted the benefits of support groups for limb loss in terms of physical ability and mobility. Research on stroke patients participating in support groups has demonstrated reductions in pain levels and disability and an increase in health-promoting behaviors, including symptom reduction.^27^ These findings are particularly relevant for PwLL, as their physical symptoms and mobility challenges significantly affect their functional capabilities and activities of daily living. This finding is supported by Asano et al,^31^ who noted perceived prosthetic mobility and social activity participation are predictors of perceived quality of life. Although not noted within this scoping review, Camacho et al^3^ found that there is decreased experience of phantom limb pain associated with social support, which is consistent with research on support groups for other medical conditions.

This review identified a positive emotional effect of peer support groups for PwLL, as evidenced by 5 studies included in this analysis. This finding is consistent with research on support groups for various medical conditions, which generally reports beneficial emotional outcomes. Specifically, depression likelihood and symptoms were found to be significantly decreased in participants of support groups for limb loss. Similarly, support groups tailored for breast cancer susceptibility gene BRCA carriers have been shown to alleviate anxiety and depression,^30^ while stroke patient support groups also reported decreased depression symptoms.^27^^,^^31^ Furthermore, when looking at upper limb versus lower limb amputation, a study Desteli et al^32^ found that upper extremity amputees had higher levels of anxiety and depression scores compared to those with lower limb amputation, which was attributed to activity restriction and decreased adjustment to prostheses. These outcomes underscore the importance of support groups in mitigating the emotional challenges faced by individuals with limb loss, whose daily tasks and overall quality of life can be significantly affected by their condition, which is supported by research from Asano et al^31^ stating that depression is a predictor of quality of life. Companionship emerged as another notable benefit, with support groups providing essential social interaction that can improve participants’ psychological adjustment and recovery. This is particularly significant for those with limb loss, as decreased mobility can limit their ability to engage with loved ones as they did before amputation. For various diagnoses, support groups show improvement in patient’s psychological adjustment and recovery by allowing opportunities for patients to socialize, view others’ perspectives, and share experiences and express feelings.^27^^,^^28^ Increased positive cognition was another positive emotional outcome of limb loss support groups, which is also observed in support groups for other medical conditions such as burn injury support groups.^29^ This suggests that, despite the unique challenges associated with limb loss, support groups can foster a more positive emotional state and support overall psychological resilience.

Learning emerged as a prevalent outcome across nearly all eligible studies included in this review. Specifically, the enhancement of self-efficacy was a recurring theme identified in limb loss support groups, a finding that aligns with outcomes observed in support groups for various other medical conditions. Notably, while participation in support groups was associated with increased self-efficacy, one study revealed that individuals engaged in self-management treatment programs exhibited even higher levels of self-efficacy.^18^ Morris and Morris^27^ demonstrated that stroke peer support group participants learned to be more self-sufficient and independent, with 10 participants agreeing with the prompts “It’s been surprising, but despite needing support from my group, I’ve also learned to be more self-sufficient” and “I believe that the group helps me to be more independent.” Additionally, another study found that participating in peer support groups cultivated skills and confidence.^29^ Self-efficacy is particularly crucial for individuals experiencing limb loss, as many aspects of their lives may initially appear unfamiliar and uncontrollable, including life with a new prosthesis. Perceived prosthetic mobility was shown to be a predictor of a participant’s quality of life.^31^ Limb loss support groups emphasize the acquisition of coping strategies, a focus that is consistent with support groups for other medical conditions. Morris and Morris^27^ discuss that one benefit of support groups for stroke patients and caregivers is the enhancement of effective coping strategies, which has a profound effect of diminishing the perceived burden from the condition. Similarly, cancer self-help groups have also been noted to promote effective coping strategies while also shaping realistic expectations regarding medical treatment and disease outcomes.^28^ Strengthening coping strategies through support groups can thus aid patients with limb loss in managing the various challenges they encounter. Furthermore, education provided by support groups after limb loss is especially important as there is a lack of education on aspects of limb loss such as phantom limb pain, as noted in the qualitative study by Camacho et al^3^ that revealed only 1 of 10 participants received information on phantom limb pain prior to experiencing the phenomenon. This emphasizes the importance of the education that is provided by support groups, as there is a deficiency in educating amputees on the many intricacies of their condition, which can be fulfilled through education dissemination in support groups.

### Implementation strategies derived using the ERIC model

Seven ERIC implementation strategies were used by 6 of the 7 studies in this review and might be adopted by health care organizations. Facilitation was the most commonly used strategy (n=6) as it enables interaction between people while also allowing problem solving and support when interaction issues arise. The ERIC model strategy to recruit, designate, and train for leadership was the least commonly described (n=1), and this may be related to the fact that most support group dynamics may not lend themselves to a hierarchical leader-based structure. The other 5 strategies were used by 3-4 studies. Involving patients or consumers and family members maintains the core focus of the support group by incorporating the perspective of PwLL in support group design and implementation. The consensus for promoting adaptability allows for local needs and innovation to help sustain the group in response to a constantly evolving health care environment. Shadowing other experts is essential for support groups to minimize reinventing the wheel and have the resources needed to develop groups within resource constraints. Finally, the ERIC model strategy to tailor strategies is vital to minimize barriers and promote facilitators for sustained member retention and superlative experience within the support group. Similar to the developers of the ERIC strategies, this review recommends selecting and adapting a combination of the abovementioned strategies to meet the needs throughout the implementation process. Furthermore, these discrete strategies should be used in conjunction with available reporting guidelines to enhance the reporting of implementation efficacy, effectiveness, and overall strategy.^13^

### Study limitations

This review was constrained by a limited number of studies, with only 7 included for analysis including only 1 randomized controlled trial. Despite approximately 2 million people living with limb loss,^1^, ^2^, ^3^ as well as over 400 registered support groups in the United States alone,^33^ there remains a paucity of research specifically addressing the structure and outcome of support groups for limb loss. Additionally, this review encompassed studies from various countries, including Australia and Canada, yet it is evident that research on international support groups for limb loss is also limited. Furthermore, the review’s eligibility criteria, which focused on articles published between 2009 and 2024, excluded potentially relevant research on support groups for limb loss published prior to 2009. Of note, this time frame also included the COVID pandemic, which could have affected the utility and prominence of support groups, especially those held in-person and the inclusion of persons at higher risk of severe disease. Furthermore, the generalizability of these results is limited as these studies tended to have a prevalence of White, younger participants with higher educational status than the general PwLL population. Therefore, results may be skewed to fitting this demographic.

### Future work

Given the current paucity of research on support groups for individuals with limb loss, several critical areas require further investigation to enhance the effectiveness of these groups. Ongoing research should focus on using this combined logic model–ERIC strategies data extraction framework to develop an implementation framework and structured methodology for developing and refining evidence-based high-quality support groups that are adaptable by context, such as upper versus lower limb loss. Further systems research in the field of limb loss support groups will facilitate implementation and sustainment of more tailored and effective support groups, thus benefiting community members, while also providing health care organizations with cost–benefit methodology to meet their community service mission.

## Conclusions

In conclusion, this scoping review highlights a significant gap in the research concerning limb loss support groups, including the frameworks for the formation, utilization, and overall efficacy of limb loss support groups. This review of 7 relevant articles from the past 15 years emphasizes that while there is a notable lack of available high-quality studies, the available evidence suggests that these support groups positively influence physical functioning and mobility, emotional well-being, and skills related to prosthesis use regardless of standardization between support groups inputs and outputs. These findings underscore the need for further research to develop a clearer understanding of the mechanisms through which support groups operate and their long-term effects on individuals with limb loss. This review provides the initial groundwork through using the novel limb loss support group logic model–ERIC strategies framework.
